# *Bullosis diabeticorum* as a differential diagnosis for limb ulcers: case report

**DOI:** 10.1590/1677-5449.202101901

**Published:** 2022-09-09

**Authors:** Vinicius Tadeu Ramos da Silva Grillo, Mayra Souza Botelho, Eloana Pasqualin Lange, Murilo Sgarbi Secanho, Paula Angeleli Bueno de Camargo, Hélio Amante Miot

**Affiliations:** 1 Universidade Estadual Paulista Júlio de Mesquita Filho – UNESP, Faculdade de Medicina, Campus de Botucatu, Botucatu, SP, Brasil.

**Keywords:** bullosis diabeticorum, blister, skin ulcer, diabetes complications, case report

## Abstract

Bullosis diabeticorum (BD) is an uncommon cutaneous manifestation of diabetes that can affect the upper limbs. It is characterized by spontaneous and painless non-inflammatory bloody blisters, which can progress to necrosis, requiring differential diagnosis to rule out other dermatological diseases, such as porphyria cutanea tarda, pseudoporphyria, epidermolysis bullosa acquisita, and pemphigoid, and vascular diseases, such as vasculitis, peripheral arterial disease, and Buerger’s disease, among others. In this report, we describe a 77-year-old male patient with poorly controlled diabetes and hypertension who presented with spontaneous onset of lesions on the upper limbs, initially with bullous characteristics, progressing to necrotic ulcers after spontaneous rupture. A biopsy revealed hyaline thickening of the dermal vessels and subcorneal bullae, consistent with a diagnosis of BD. After smoking cessation and optimization of glycemia control combined with topical corticosteroid therapy, the condition improved and lesions began to heal. This presentation of BD involving the upper limbs is rare, requiring differential diagnosis to rule out other cutaneous and vascular lesions.

## INTRODUCTION

*Bullosis diabeticorum* is an uncommon cutaneous manifestation of diabetes. It is characterized by recurrent non-inflammatory, painful blisters with spontaneous onset and self-limiting course.^1^^,^^2^ It occurs in 0.5 to 2% of diabetics, with a predilection for male patients and greater recurrence in lower limbs, particularly the feet. Although rarer, it can also affect the upper limbs, hands, and fingers.^1^^,^^3^

We report the case of a patient with ulcers of the upper limbs diagnosed with *bullosis diabeticorum*. Since this condition is uncommon in this location, with few cases reported in the literature, the objective of this article is to highlight the importance of knowledge of the possible differential diagnoses of cutaneous lesions in diabetic patients. The protocol was approved by the Ethics Committee at our institution (decision number 4.977.446).

## CASE REPORT

A 77-year-old, white, male patient presented at the emergency room with painless lesions on the fingers, described by the initial care team as ulcerations with areas of necrosis. He was referred to a tertiary hospital service for investigation of suspected peripheral arterial disease by the vascular surgery team. He reported that blisters had appeared on his upper limbs spontaneously 3 months previously, bursting spontaneously 1 to 2 days afterwards, and developing into necrotic ulcers. He also stated that during this time some blisters had healed and others had appeared. His medical history included systemic arterial hypertension, uncontrolled type 2 Diabetes mellitus, diagnosed more than 20 years previously, an active, long-term, smoking habit, with a 20 pack-years tobacco load, and diabetic neuropathy. He was taking the following medications irregularly: 850 mg/day of metformin, 30 mg/day of gliclazide, 35 units in the morning and 35 units at night of Neutral Protamine Hagedorn (NPH) insulin, 100 mg/day of acetylsalicylic acid (ASA), 50 mg of cilostazol every 12 hours, 20 mg of enalapril every 12 hours, 20 mg/day of simvastatin, and 50 mg/day of sertraline.

**Figure 1:**
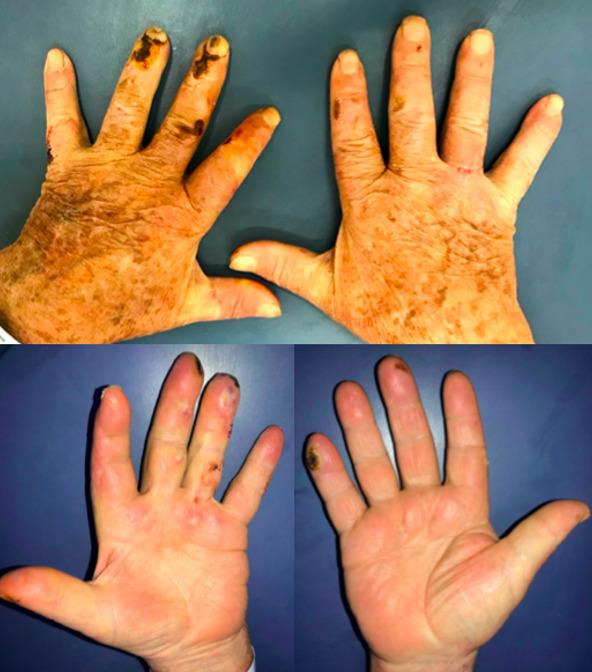
Hyaline vesicles on the third and fourth left fingers and exulcerations with necrotic scabs on the dorsal and distal fingers (December/2020).

Physical examination revealed three blisters with taut, thick surfaces, one on the palm of the left hand and the others on the third and fourth fingers, each measuring approximately 0.8 cm in diameter. The patient also had ulcerated lesions with necrotic centered scabs on the dorsal surfaces of the second, fourth, and fifth fingers of the left hand and the fifth finger of the right hand, all around 1.5 x 1 cm in size (Figure 1). The vascular examination found a good radial pulse and palpable ulnar pulse on the right. On the left, both the radial and ulnar pulses were strong. There were no trophic ulcers on lower limbs, and femoral, popliteal, and posterior tibial pulses were palpable on the left and femoral and popliteal pulses were palpable on the right. Laboratory tests were ordered and the results for full blood count, renal function, uric acid, electrolytes, and liver function and injury indicators were all normal. Uncontrolled diabetes was revealed by 11.7% glycated hemoglobin, well above the target for diabetics (< 7%).

Specialist clinical assessment ruled out pathologies of a vascular origin and the patient was referred for assessment by the Dermatology team, which raised a hypothesis of *bullosis diabeticorum*, a rare variant involving the hands, and pseudoporphyria.

**Figure 2:**
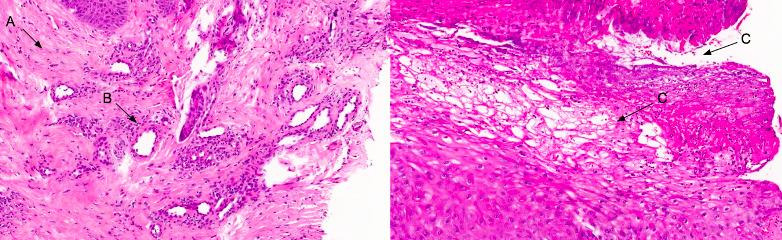
Histopathology images of bullous lesions of the patient’s hand, showing: **(A)** eosinophils and neutrophils; **(B)** perivascular fibrinoid deposits; and **(C)** high intraepidermal cleavage.

Review of the patient’s medical records showed that the patient had already been seen by a dermatology service because of the lesions described above, but had failed to maintain follow-up 2 years previously. During follow-up, the patient had had a biopsy of a blister of the fourth finger and the specimen had been sent to a pathology laboratory, which had reported hyaline thickening of dermal vessels and subcorneal bullae, compatible with a diagnosis of *bullosis diabeticorum* (Figure 2).

The patient was given recommendations on improving lifestyle habits and quitting smoking and was referred to the endocrinology service for specialist diabetes care. Dressings with collagenase were prescribed for 1 month, and, after reassessment showed improvement of the lesions, he started to use a cream prepared at a compounding pharmacy containing 0.1% betamethasone, 12% urea, 2% silicone oil, and the base cream.

**Figure 3:**
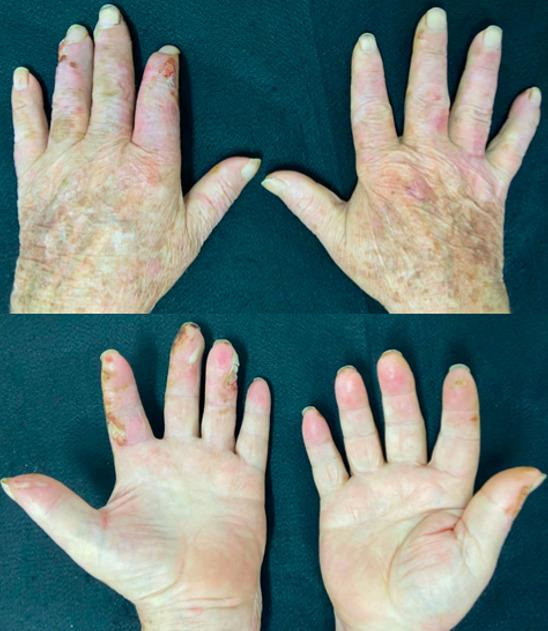
The same patient in April of 2021, with vesicles on the right thumb and a smaller quantity of exulcerations with fibrinoid base on the fingers.

At a follow-up consultation 3 months later, the patient was tobacco abstinent, his glycemic control had improved, and the lesions were healing (Figure 3).

## DISCUSSION

Diabetes mellitus is a chronic disease with high prevalence, morbidity, and mortality. According to estimates from the International Diabetes Federation (IDF), 463 million people (9.3% of the global adult population) are affected by the disease, with a tendency for incidence to increase progressively over the coming decades. Its many different associated complications make this disorder an important risk factor for premature mortality and impaired quality of life, with substantial impacts on patients and healthcare systems.^4^

Cutaneous manifestations can be found in a significant proportion of diabetic patients, because the elevated serum glucose levels stimulate cellular damage and suppress regeneration of the epidermis either directly or indirectly via multiple pathways. Indirectly, hyperglycemia produces advanced glycation products that react with type 1 collagen and with epidermal growth factor receptor, suppressing cellular regeneration. High levels of serum glucose also lead to vasodilation dysfunction through inhibition of nitric oxide molecules, thereby dysregulating the blood supply to the skin. Hyperglycemia thus affects keratinocytes and fibroblast activity, causing changes to protein synthesis, proliferation, and migration, which are essential processes for maintenance of cutaneous integrity.^2^

The most common dermatoses in diabetics include xerosis cutis, acanthosis nigricans, acrochordons, diabetic dermopathy, and infections and ulcers of the diabetic foot. Xerosis, or dry skin, is caused by dysfunction of cutaneous integrity maintenance and makes itching and secondary skin infections more likely. In addition to being one of the primary cutaneous manifestations of diabetes, acanthosis nigricans can be present in up to 74% of obese patients who only have hyperinsulinemia, is characterized by chestnut plaques with a velvet textured surface, and is generally found on the neck, the groin, or the armpits. Sessile or pedunculated, chestnut-colored papules called acrochordons (or skin tags) often emerge from areas of acanthosis. In turn, diabetic dermopathy presents as macules, papules, or pretibial atrophic plaques that generally heal spontaneously within 1 to 2 years, leaving an atrophic and hypopigmented area. Although they have low morbidity, these skin conditions can have considerable esthetic impact on patients and treatment includes diabetes control.^5^^,^^6^

The most commonly associated skin infections are candidiasis, dermatophytosis, and bacterial infections, each of which has its own group of pathologies. Generally, these conditions are more common and more severe among diabetics than in the general population, since diabetics have white blood cell dysfunction secondary to the elevated glucose levels. Of all these infections, diabetic foot ulcers merit special mention because of their considerable associated morbidity. Both emergence and the difficulty of resolving them are related to diabetic neuropathy and the immunological and regenerative skin dysfunctions described above.^5^^,^^6^

Moreover, diabetes is an important risk factor for peripheral arterial disease, which, although predominantly observed in the lower limbs, can also involve upper limbs, with formation of ulcers.^7^

*Bullosis diabeticorum* is one of the cutaneous complications seen in diabetics. It was first described by Kramer in 1930 and later characterized by Cantwell and Martz in 1967.^3^ It is a disease that primarily affects long-term diabetics, but has also been described as a first presentation in patients with glucose intolerance.^8^ Its pathophysiology has not been fully explained, but appears to be multifactorial. The predominantly acral location of lesions led to the hypothesis that they could be trauma related. Additionally, a considerable proportion of patients also have diabetic kidney disease and diabetic neuropathy, which suggests that microangiopathy could possible play a role in the condition.^9^ It has been demonstrated that diabetic patients are more likely to form suction-induced blisters.^10^

The disease has higher prevalence among men aged 17 to 80 years, with a mean age of 55 years, and in uncontrolled diabetes.^9^ It has abrupt onset, usually at night, with no link with a history of prior trauma, and spontaneous resolution in 2 to 6 weeks.^11^ It is characterized by taut vesicles or blisters with no inflammatory signs, i.e., without a characteristic erythematous base, with serous content, sometimes hemorrhagic, and, if there is secondary infection, with pus. Diagnosis can be achieved by ruling out differential diagnoses and by correlation with pathology findings. Pathology may reveal intraepidermal or subepidermal bullae and direct immunofluorescence is negative.^9^

Differential diagnoses include bullous pemphigoid, epidermolysis bullosa acquisita, porphyria cutanea tarda, and drug-induced bullous dermatosis.^3^

The bullae seen in porphyria cutanea tarda and pseudoporphyria are generally smaller than 1 cm and prefer the hands to the feet and ankles. In addition to porphyria cutanea tarda having other clinical cutaneous findings, such as hypertrichosis, it also exhibits specific patterns under immunofluorescence and accumulation of porphyrins in urine and feces. Pseudoporphyria has similar histopathology to porphyria, but does not involve elevated porphyrins. Pseudoporphyria is not uncommon in patients with diabetes, because they can develop complications such as chronic renal failure and atherosclerotic cardiovascular disease and may be on dialysis and/or diuretics, which trigger this pathology. It is thus necessary to rule out these complications in diabetic patients to indicate that a diagnosis of pseudoporphyria is unlikely.^9^^,^^10^

Distal extremities are also common sites for erythema multiforme and fixed drug rashes, but such blisters generally form over an inflammatory base. Acquired epidermolysis bullosa and localized bullous pemphigoid are differentiated from *bullosis diabeticorum* by histological findings and by direct immunofluorescence and also by their preference for sites of trauma and friction in the case of epidermolysis bullosa acquisita. If there is a surrounding skin inflammation with erythema, heat, and sensitivity, the possibility of bullous cellulitis should also be considered.^9^^,^^10^

Treatment involves care such as local dressings or aspiration of the liquid content, which may be necessary in cases with large blisters. This should be done under aseptic conditions and exeresis of the cutaneous envelope is not recommended, since it can provide good coverage of the site.^12^ Dressings are applied to reduce the risk of trauma and secondary infections. The risk of osteomyelitis makes serial assessments necessary.^3^ In advanced cases, with advanced local or systemic infections, debridement and negative pressure dressings can be employed with good results. Clinical care should be based on control of the underlying comorbidity and rigorous surveillance of foot and hand traumas.^13^

## CONCLUSIONS

This case report stands out because of the rare presentation involving the upper limbs and because of the importance of knowledge of the main differential diagnoses such as bullous pemphigoid, epidermolysis bullosa, and porphyria cutanea tarda, and vascular diseases such as vasculitis, peripheral arterial disease, and Buerger’s disease, among others.
