# Supplementary material for: Bullosis diabeticorum as a differential diagnosis for limb ulcers: case report
Source: J Vasc Bras. 2022 Sep 9;21:e20210190. doi: 10.1590/1677-5449.202101901 (PMC9477478; doi:10.1590/1677-5449.202101901)
Supplement: Translation

# Translation: *Bullosis diabeticorum* como diagnóstico diferencial de úlcera de membros: relato de caso

**DOI:** 10.1590/1677-5449.202101902

**Authors:** Vinicius Tadeu Ramos da Silva Grillo, Mayra Souza Botelho, Eloana Pasqualin Lange, Murilo Sgarbi Secanho, Paula Angeleli Bueno de Camargo, Hélio Amante Miot

**Affiliations:** 1 Universidade Estadual Paulista Júlio de Mesquita Filho – UNESP, Faculdade de Medicina, Campus de Botucatu, Botucatu, SP, Brasil.

**Keywords:** bullosis diabeticorum, vesícula, úlcera cutânea, complicações do diabetes, relato de caso

## Abstract

A *bullosis diabeticorum* (BD) é uma manifestação cutânea infrequente do diabetes e pode afetar os membros superiores. Caracteriza-se pelo aparecimento espontâneo e indolor de flictenas não inflamatórias de aspecto sanguinolento, que podem evoluir para necrose, requerendo que se faça diagnóstico diferencial com doenças dermatológicas, como porfiria cutânea tarda, pseudoporfiria, epidermólise bolhosa adquirida e penfigoide bolhoso, e doenças vasculares, como vasculites, doença arterial periférica, doença de Buerger, entre outras. Neste relato, descreve-se o caso de um paciente masculino de 77 anos, hipertenso e diabético mal controlado, apresentando aparecimento espontâneo de lesões bolhosas de membros superiores, que evoluíram, após ruptura espontânea, para ulcerosas necróticas. A biópsia da lesão diagnosticou a presença de espessamento hialino de vasos dérmicos e de bolhas subcórneas, compatíveis com o diagnóstico de BD. Após a interrupção do tabagismo e a otimização do controle glicêmico associado a corticoterapia tópica, houve melhora, com cicatrização das lesões. A apresentação da BD em membros superiores é mais rara, sendo necessário o diagnóstico diferencial com outras lesões cutâneas e vasculares.

## INTRODUÇÃO

A *bullosis diabeticorum* é uma manifestação cutânea infrequente do diabetes. Caracteriza-se por bolhas não inflamatórias, indolores, de aparecimento espontâneo e de curso autolimitado e recorrente^1^^,^^2^. Ela ocorre em 0,5 a 2% dos diabéticos, com maior acometimento em pacientes do sexo masculino, sendo mais recorrente nos membros inferiores, sobretudo nos pés. Apesar de mais raramente, também pode afetar os membros superiores, mãos e quirodáctilos^1^^,^^3^.

Relatamos o caso de um paciente com quadro de úlceras em membros superiores e diagnóstico de *bullosis diabeticorum*. Dado que se trata de uma condição incomum nessa localização, com poucos casos relatados na literatura, este artigo objetiva ressaltar a importância de se atentar aos possíveis diagnósticos diferenciais das lesões cutâneas no paciente diabético. O protocolo foi aprovado pelo Comitê de Ética da nossa instituição (parecer número 4.977.446).

## RELATO DE CASO

Um paciente de 77 anos, do sexo masculino e branco procurou o pronto-socorro por conta de lesões indolores nos dedos das mãos, referidas pela equipe de atendimento inicial como ulcerações com áreas de necrose. Devido à suspeita de doença arterial periférica, foi encaminhado ao serviço hospitalar terciário para avaliação da área de Cirurgia Vascular. O paciente referia que há 3 meses iniciou aparecimento espontâneo de lesões bolhosas nos membros superiores, que se romperam espontaneamente em 1 a 2 dias, evoluindo para lesões ulcerosas necróticas. Também relatou que, nesse intervalo, houve cicatrização de algumas lesões e surgimento de novas. Ele apresentava histórico de hipertensão arterial sistêmica, diabetes melito tipo 2 descompensado, diagnosticado há mais de 20 anos, e tabagismo ativo de longa data, com carga tabágica de 20 anos-maço, além de neuropatia diabética. Fazia uso irregular das seguintes medicações: 850 mg/dia de metformina, 30 mg/dia de gliclazida, 35 unidades pela manhã e 35 à noite de insulina NPH (do inglês *Neutral Protamine Hegedorn*), 100 mg/dia de ácido acetilsalicílico (AAS), 50 mg de 12/12 h de cilostazol, 20 mg de 12/12 h de enalapril, 20 mg/dia de sinvastatina e 50 mg/dia de sertralina.

Ao exame físico, apresentava três bolhas de superfície tensa e espessa, sendo uma na palma da mão esquerda e as outras no terceiro e quarto quirodáctilos, medindo aproximadamente 0,8 cm de diâmetro cada. Além disso, havia lesões ulceradas com crostas necróticas centrais no dorso dos segundo, quarto e quinto dedos da mão esquerda e do quinto dedo da mão direita, todas com cerca de 1,5 x 1 cm (Figura 1). Quanto ao exame vascular, apresentava pulso radial bom à direita e pulso ulnar palpável. À esquerda, ambos os pulsos radial e ulnar estavam bons e amplos. Não havia lesões tróficas nos membros inferiores, com pulsos femoral, poplíteo e tibial posterior palpáveis à esquerda e femoral e poplíteo palpáveis à direita. Foram realizados exames laboratoriais, com hemograma, função renal, ácido úrico, eletrólitos, função e lesão hepática normais. Foi constatado diabetes descompensado pela presença de hemoglobina glicada de 11,7%, muito acima da meta estabelecida para o diabético (< 7%).

**Figura 1:** Vesículas hialinas nos terceiro e quarto quirodáctilos esquerdos e exulcerações com crostas necróticas em dorso e porções distais dos dedos (dezembro/2020).

Após avaliação clínica especializada, foi excluída patologia de origem vascular, e o paciente foi encaminhado para avaliação da equipe de Dermatologia, que aventou a hipótese de *bullosis diabeticorum*, variante rara das mãos, e pseudoporfiria.

Em revisão de prontuário, foi evidenciado que o paciente já havia sido avaliado pela Dermatologia devido às lesões supracitadas, mas havia perdido seguimento há 2 anos. Durante o acompanhamento, o paciente foi submetido a biópsia de lesão bolhosa do quarto quirodáctilo, e o material foi encaminhado para a anatomopatologia, que demonstrou a presença de espessamento hialino de vasos dérmicos e de bolhas subcórneas, compatíveis com o diagnóstico de *bullosis diabeticorum* (Figura 2).

**Figura 2:** Imagens histopatológicas das lesões bolhosas da mão do paciente, mostrando: **(A)** presença de eosinófilos e neutrófilos; **(B)** depósito fibrinoide perivascular; e **(C)** clivagem intraepidérmica alta.

O paciente foi orientado quanto à melhora dos hábitos de vida e cessação do tabagismo, tendo sido encaminhado para atendimento especializado do diabetes com a Endocrinologia. Foram prescritos curativos com colagenase por 1 mês, e, após reavaliação e melhora das lesões, passou a utilizar creme manipulado com associação de betametasona 0,1%, ureia 12%, óleo de silicone 2% e creme-base.

Na consulta de retorno, após 3 meses, o paciente apresentava-se abstinente do tabaco, com melhora do controle glicêmico e cicatrização das lesões (Figura 3).

**Figura 3:** Mesmo paciente em abril de 2021, com vesículas no polegar direito e menor quantidade de exulcerações nos quirodáctilos com fundo fibrinoide.

## DISCUSSÃO

O diabetes melito é uma doença crônica com prevalência e morbimortalidade altas. Conforme estimativas da Federação Internacional de Diabetes (IDF), 463 milhões de pessoas (9,3% da população mundial adulta) são afetadas pela doença, com tendência de aumento progressivo da incidência nas próximas décadas. Devido às diversas complicações associadas, essa enfermidade representa um importante fator de risco para mortalidade prematura e redução da qualidade de vida, gerando impactos substanciais nos pacientes e nos sistemas de saúde^4^.

As manifestações cutâneas podem ser encontradas em parcela significativa dos pacientes diabéticos, pois o nível elevado de glicose sérica estimula o dano celular e suprime a regeneração da epiderme de maneira direta e indireta, por meio de múltiplas vias. Indiretamente, a hiperglicemia origina produtos da glicação avançada que reagem com o colágeno tipo 1 e com o receptor do fator de crescimento epidérmico, suprimindo a regeneração celular. Altos níveis de glicose sérica também levam à disfunção na vasodilatação por meio da inibição das moléculas de óxido nítrico, desregulando, assim, o suprimento sanguíneo cutâneo. Dessa forma, a hiperglicemia afeta os queratinócitos e as atividades dos fibroblastos, causando alterações na síntese, proliferação e migração de proteínas, processos essenciais para manutenção da integridade cutânea^2^.

Entre as dermatoses mais comuns nos diabéticos, destacam-se a xerose cutânea, acantose nigricans, acrocórdons, dermopatia diabética, infecções e úlceras do pé diabético. O ressecamento cutâneo, denominado xerose, é explicado pelas disfunções na manutenção da integridade cutânea e favorece o estabelecimento de prurido e infecções cutâneas secundárias. A acantose nigricans, além de ser uma das principais manifestações cutâneas do diabetes, pode estar presente em 74% dos pacientes obesos que possuam apenas hiperinsulinemia e é caracterizada por placa acastanhada de superfície aveludada ao toque presente geralmente no pescoço, na região inguinal ou nas axilas. Frequentemente, sobre tal alteração, surgem pápulas acastanhadas sésseis ou pedunculadas chamadas acrocórdons. Já a dermatopatia diabética se apresenta como máculas, pápulas ou placas atróficas pré-tibiais que geralmente cicatrizam espontaneamente em 1 a 2 anos, deixando área atrófica e hipopigmentada no local. Tais condições dermatológicas, apesar de baixa taxa de morbidade, têm impacto estético importante sobre os pacientes, e, entre seu tratamento, inclui-se o controle do diabetes^5^^,^^6^.

As infecções cutâneas mais comumente associadas são as candidoses, dermatofitoses e infecções bacterianas, cada qual com seu grupo de patologias. Geralmente esses quadros são mais comuns e mais graves nos diabéticos do que na população em geral, pois neles há uma disfunção dos glóbulos brancos secundária a níveis elevados de glicose. Vale ressaltar, entre as infecções, as úlceras do pé diabético, que necessitam de destaque pela morbidade relacionada. Seu surgimento e sua difícil resolução estão relacionados com a neuropatia diabética e a disfunção imunológica e regenerativa da pele descritas acima^5^^,^^6^.

Ademais, o diabetes é importante fator de risco para doença arterial periférica, cujo acometimento, apesar de ser predominantemente observado nos membros inferiores, também pode acometer os membros superiores, com formação de úlceras^7^.

A *bullosis diabeticorum* é uma das complicações cutâneas do diabético. Foi relatada primeiramente por Kramer em 1930 e posteriormente caracterizada por Cantwell e Martz em 1967^3^. É uma doença que afeta principalmente diabéticos de longa data, porém já foi descrita como primeira apresentação em pacientes com intolerância à glicose^8^. A sua fisiopatologia ainda não está esclarecida, mas parece ser multifatorial. A predominância da localização acral dessas lesões levou à hipótese de que possam estar relacionadas a trauma. Além disso, boa parte dos pacientes afetados apresenta concomitantemente doença renal do diabetes e neuropatia diabética, o que sugere possível papel da microangiopatia na etiologia dessa enfermidade^9^. Já foi demonstrado também que pacientes diabéticos têm maior chance de formação de bolhas induzidas por sucção^10^.

Essa doença ocorre com maior prevalência em homens, entre 17 e 80 anos, com idade média de 55 anos e diabetes descontrolado^9^. Desenvolve-se abruptamente, em geral à noite, sem história de trauma prévio, com resolução espontânea, em 2 a 6 semanas^11^. Caracteriza-se por vesículas ou bolhas tensas, sem sinais inflamatórios, ou seja, sem base eritematosa característica, com conteúdo seroso, por vezes hemorrágico, e, caso haja infecção secundária, pus. O diagnóstico pode ser obtido a partir da exclusão de diagnósticos diferenciais e correlação clínico-histopatológica. Na histopatologia, podemos observar bolhas intraepidérmicas ou subepidérmicas e a imunofluorescência direta encontra-se negativa^9^.

Os diagnósticos diferenciais incluem penfigoide bolhoso, epidermólise bolhosa adquirida, porfiria cutânea tarda e lesões bolhosas induzidas por drogas^3^.

Na porfiria cutânea tarda e na pseudoporfiria, as bolhas são geralmente menores que 1 centímetro e favorecem as mãos em vez dos pés e tornozelos. Além da porfiria cutânea tarda possuir outros achados clínicos cutâneos, como hipertricose, ela apresenta padrões específicos na imunofluorescência e acúmulos de porfirinas na urina e nas fezes. A pseudoporfiria possui histopatologia semelhante à porfiria, mas não possui elevação de porfirinas. A pseudoporfiria não é incomum em pacientes com diabetes, pois eles podem desenvolver complicações, incluindo insuficiência renal crônica e doença cardiovascular aterosclerótica e, portanto, podem estar recebendo diálise e/ou diuréticos, que são desencadeadores da patologia. Dessa forma, é necessário descartar tais complicações nos pacientes diabéticos para desfavorecer o diagnóstico de pseudoporfiria^9^^,^^10^.

A extremidade distal também é um local comum para eritema multiforme e erupções medicamentosas fixas, mas as bolhas geralmente se desenvolvem em uma base inflamatória. A epidermólise bolhosa adquirida e o penfigoide bolhoso localizado são diferenciados da *bullosis diabeticorum* pelo exame histológico e pela imunofluorescência direta, bem como pela preferência das lesões pelos locais de trauma e fricção em pacientes com epidermólise bolhosa adquirida. Se houver uma pele inflamatória circundante com eritema, calor e sensibilidade, a possibilidade de celulite bolhosa também deve ser considerada^9^^,^^10^.

O tratamento envolve cuidados como curativo local ou aspiração do conteúdo líquido, que pode ser necessária em casos de bolhas volumosas. Ela deve ser realizada de maneira asséptica, e não é indicada a exérese do envelope cutâneo, que pode fornecer uma boa cobertura local^12^. Curativos são realizados para diminuir o risco de trauma e infecções secundárias. Devido ao risco de osteomielite, avaliações seriadas são necessárias^3^. Em casos avançados, com presença de infecção avançada local ou sistêmica, pode ser indicada a realização de desbridamento e curativo com terapia de pressão negativa, com bom resultado. O seguimento clínico deve ser pautado em controle da comorbidade de base e vigilância rigorosa de traumas em pés e mãos^13^.

## CONCLUSÕES

Este relato de caso se destaca pela apresentação mais rara em membros superiores e pela importância do conhecimento dos principais diagnósticos diferenciais como penfigoide bolhoso, epidermólise bolhosa, porfiria cutânea tarda e doenças vasculares, como vasculites, doença arterial periférica, doença de Buerger, entre outras.
